# Purpose in Life and Estimated Type 2 Diabetes Risk: Cross-Sectional Associations Across Three Validated Risk Scores in 93,077 Spanish Working Adults

**DOI:** 10.3390/medsci14010113

**Published:** 2026-02-26

**Authors:** Pilar García Pertegaz, Pedro Juan Tárraga López, Irene Coll Campayo, Carla Busquets-Cortés, Ángel Arturo López-González, José Ignacio Ramírez-Manent

**Affiliations:** 1 Quirón Salud Palma Planas Hospital, 07014 Palma, Spain; pilarpertegaz@gmail.com; 2 Faculty of Medicine, University of Castilla La Mancha (UCLM), 02008 Albacete, Spain; pjtarraga@sescam.jccm.es; 3 ADEMA-University School, University of the Balearic Islands,07009 Palma, Spain; i.coll@eua.edu.es (I.C.C.); c.busquets@eua.edu.es (C.B.-C.); joseignacio.ramirez@ibsalut.es (J.I.R.-M.); 4 Faculty of Medicine, University of the Balearic Islands, 07120 Palma, Spain

**Keywords:** purpose in life, type 2 diabetes risk, psychosocial factors, diabetes risk scores, cardiometabolic health, occupational population

## Abstract

**Background**: Psychosocial well-being has been increasingly recognized as a relevant factor in cardiometabolic health; however, evidence linking Purpose in Life with type 2 diabetes risk across validated prediction tools remains limited. This study examined the association between Purpose in Life and estimated diabetes risk using three established risk scores. **Methods**: A cross-sectional analysis was performed in 93,077 Spanish working adults aged 18–69 years participating in routine occupational health assessments. Purpose in Life was measured with the 10-item Purpose in Life scale and categorized into high, moderate, and low levels. Estimated type 2 diabetes risk was evaluated using QDScore, FINDRISC, and CANRISK. Multivariable logistic regression models were applied to calculate odds ratios (ORs) and 95% confidence intervals (CIs), adjusting for age, sex, occupational social class, smoking status, dietary pattern, physical activity, and body mass index. **Results**: Lower levels of Purpose in Life were consistently associated with greater likelihood of high estimated diabetes risk across all three instruments. Compared with participants reporting high Purpose in Life, those with low Purpose in Life showed increased odds of high-risk classification for QDScore (OR 2.38; 95% CI 2.19–2.57), FINDRISC (OR 2.49; 95% CI 2.08–2.89), and CANRISK (OR 2.79; 95% CI 2.50–3.09). Clear dose–response patterns were observed across Purpose in Life categories, and associations were similar in men and women as well as across lifestyle strata. **Conclusions**: Reduced Purpose in Life is strongly associated with higher estimated type 2 diabetes risk across multiple validated screening tools. Although causal direction cannot be inferred from this cross-sectional design, these findings suggest that psychosocial dimensions may provide complementary information for cardiometabolic risk assessment and prevention strategies.

## 1. Introduction

Type 2 diabetes mellitus is a leading global health concern in the 21st century, marked by a steadily rising prevalence, considerable cardiovascular complications, and substantial economic impact on healthcare systems. Current international estimates indicate that more than 500 million adults worldwide are living with diabetes, and projections suggest further increases driven by demographic aging, physical inactivity, and worsening metabolic risk profiles [1,2]. In this context, identifying individuals at increased risk at an early stage remains a cornerstone of preventive medicine and occupational health practice.

Several validated, non-invasive prediction tools have been introduced to facilitate early risk assessment in both clinical and community settings. Risk scores such as QDScore, FINDRISC, and CANRISK combine demographic, anthropometric, clinical, and behavioral variables to estimate future type 2 diabetes probability without the need for immediate biochemical testing [3,4,5,6]. Their use enables rapid cardiometabolic risk stratification and supports targeted preventive interventions.

In addition to traditional metabolic and lifestyle determinants, psychosocial factors are increasingly recognized as relevant contributors to cardiometabolic health. Indicators of psychological well-being, including optimism and perceived life meaning, have been associated with lower cardiovascular incidence, reduced mortality, and more favorable biological risk patterns [7,8,9,10]. Within this framework, Purpose in Life—conceptualized as the degree to which individuals experience their lives as meaningful, goal-oriented, and aligned with personally valued aims—has attracted growing research interest. As a core component of eudaimonic well-being, Purpose in Life reflects existential meaning, motivational direction, and future-oriented engagement, and has been proposed as a psychological resource that promotes resilience and healthy aging. The Purpose in Life test (PIL), originally developed in the field of existential psychology, has been widely used in health and behavioral sciences to evaluate life meaning and motivational functioning. Studies employing the PIL and related instruments have linked higher levels of purpose to better mental health, improved quality of life, healthier behaviors, cognitive preservation, reduced cardiovascular risk, and lower overall mortality [11].

Longitudinal cohort evidence further indicates that individuals reporting stronger life purpose experience lower rates of all-cause mortality, fewer cardiovascular events, and reduced functional decline over time [12]. These associations may be partially explained by behavioral mechanisms, as individuals with greater purpose are more likely to engage in regular physical activity, adhere to healthier dietary patterns, and avoid tobacco use [13]. Moreover, biological pathways have been suggested, including attenuated chronic stress responses, more favorable neuroendocrine functioning, and lower systemic inflammatory activity—processes closely related to insulin resistance and metabolic dysregulation [14,15,16].

Despite the expanding literature on Purpose in Life and major health outcomes, its association with estimated type 2 diabetes risk as determined by validated prediction scores has not been extensively examined, particularly in large working populations. Much of the existing research has focused on mortality or cardiovascular endpoints, whereas fewer studies have evaluated psychosocial constructs in relation to diabetes risk stratification tools commonly applied in preventive settings. Determining whether Purpose in Life correlates with established diabetes risk scores may contribute to more integrative cardiometabolic risk assessment approaches.

Therefore, the present study aimed to investigate the relationship between Purpose in Life and estimated type 2 diabetes risk using three validated instruments—QDScore, FINDRISC, and CANRISK—in a large occupational cohort of Spanish workers. We hypothesized that lower levels of Purpose in Life would be associated with higher diabetes risk across all instruments and that a graded association would persist after adjustment for sociodemographic and lifestyle covariates. Given the cross-sectional design, the analyses were intended to evaluate associations rather than infer causality.

## 2. Methods

### 2.1. Study Design and Population

This cross-sectional analysis was conducted within a large occupational health cohort of Spanish workers who underwent routine workplace medical examinations between January 2019 and December 2023. Health assessments were performed in accredited occupational health centers using standardized clinical and laboratory procedures, in accordance with STROBE recommendations for observational studies.

During the study period, 95,049 workers were evaluated. After applying predefined eligibility criteria, 93,077 participants with complete data for psychosocial assessment, diabetes risk score calculation, and covariates were included in the final analytical sample.

The cohort comprised active employees from multiple economic sectors across Spain, including industrial, service, administrative, and manual occupations. Occupational social class was classified according to the Spanish National Classification of Economic Activities (CNAE-11) and grouped into three categories following Spanish Society of Epidemiology criteria.

### 2.2. Inclusion and Exclusion Criteria

**Figure 1:**
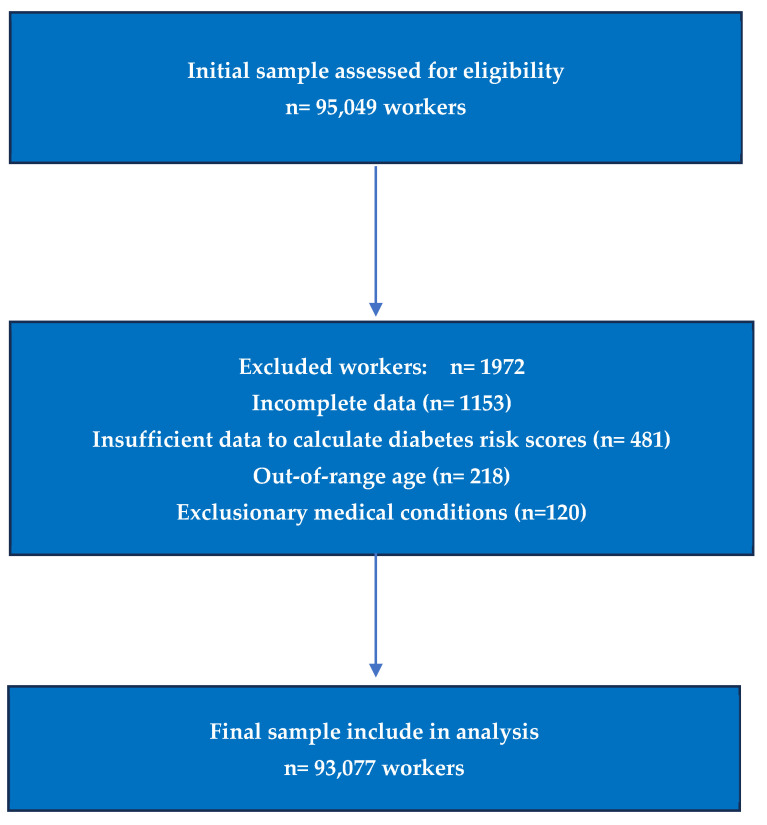
Flow chart of the participants.

Participants were eligible if they were active employees aged 18–69 years with complete sociodemographic, clinical, laboratory, and questionnaire data required for diabetes risk score computation and covariate adjustment. Exclusion criteria comprised missing components of any diabetes risk score, incomplete Purpose in Life assessment, pregnancy, severe chronic conditions affecting metabolic evaluation, and inconsistent or duplicate records. These criteria are consistent with those applied in previous large occupational cardiometabolic studies conducted in Spanish working populations (Figure 1).

### 2.3. Clinical and Anthropometric Assessment

All clinical and anthropometric measurements were performed by trained healthcare professionals using standardized procedures and calibrated equipment. Body weight and height were measured with participants wearing light clothing and no shoes, and body mass index (BMI) was calculated as kg/m^2^. Waist and hip circumferences were assessed using a non-elastic measuring tape at standardized anatomical landmarks.

Blood pressure was recorded after a 5 min seated rest using validated automated sphygmomanometers. Fasting venous blood samples were collected and analyzed in certified laboratories using enzymatic methods to determine glucose and lipid parameters.

Anthropometric procedures followed World Health Organization recommendations [17], and blood pressure assessment adhered to European Society of Cardiology/European Society of Hypertension guidelines [18]. These measurements were used both as covariates and, where applicable, as components of the diabetes risk scores (Table 1).

### 2.4. Assessment of Type 2 Diabetes Risk Scores

Estimated type 2 diabetes risk was evaluated using three validated non-invasive instruments integrating demographic, anthropometric, clinical, and lifestyle information:

- QDScore, a multivariable algorithm developed in UK primary care populations to estimate future diabetes risk [19].
- FINDRISC, a questionnaire-based tool validated in European populations to assess 10-year diabetes risk [20].
- CANRISK, an adaptation of FINDRISC calibrated for North American populations [21].

High-risk categories were defined according to established cut-off values from the original validation studies. The application of multiple independent risk scores enabled assessment of cross-instrument consistency and reduced reliance on any single predictive framework. The number and proportion of participants exceeding each threshold are presented in Table 2.

### 2.5. Sociodemographic and Occupational Variables

Sociodemographic characteristics included age, sex, and occupational social class. Occupational class was classified according to the Spanish National Classification of Economic Activities (CNAE-11) and grouped into three categories following Spanish Society of Epidemiology criteria, an approach widely used in occupational and population health research in Spain [22].

### 2.6. Lifestyle Factors

Dietary adherence was assessed using the 14-item Mediterranean Diet Adherence Screener (MEDAS-14), validated in Spanish populations [23]. Scores range from 0 to 14, with values ≥ 9 indicating good adherence. For multivariable analyses, adherence was treated as a categorical variable (adequate vs. suboptimal).

Physical activity was evaluated using the International Physical Activity Questionnaire–Short Form (IPAQ-SF), which captures activity performed during the previous week across intensity domains [24]. Responses were converted into MET-minutes/week and categorized into low, moderate, or high activity levels according to standard scoring criteria.

Smoking status was self-reported and coded dichotomously as current smoker versus non-smoker [25]. Alcohol consumption was not systematically recorded in the occupational health protocol and therefore was not included in the analyses.

### 2.7. Purpose in Life Assessment

Purpose in Life was measured using the 10-item Purpose in Life scale (PIL-10), with items rated on a 7-point Likert scale (total score range: 10–70). Higher scores reflect greater perceived meaning and goal orientation. Internal consistency in this cohort was high (Cronbach’s α = 0.89; McDonald’s ω = 0.91).

When a single item was missing, the participant-specific mean of completed items was imputed; individuals missing two or more items were excluded from analyses involving the PIL-10. Total scores were categorized into low, moderate, and high levels using the predefined classification scheme [26].

### 2.8. Statistical Analysis

Continuous variables are reported as means and standard deviations, and categorical variables as frequencies and percentages. Group comparisons were conducted using analysis of variance or chi-square tests as appropriate. Cohen’s d was calculated to quantify sex-related differences in continuous variables.

Multivariable logistic regression models were fitted to estimate adjusted odds ratios (ORs) and 95% confidence intervals (CIs) for high diabetes risk according to QDScore, FINDRISC, and CANRISK across Purpose in Life categories, using high Purpose in Life as the reference group. Models were adjusted for age, sex, occupational social class, smoking status, Mediterranean diet adherence, physical activity, and BMI. High diabetes risk was modeled as a binary outcome, and separate models were constructed for each risk score. All covariates were entered simultaneously using a forced-entry approach.

Covariates were selected a priori based on epidemiological evidence and a conceptual causal framework. Additional crude analyses based on aggregated subgroup data were performed to evaluate linear trends and potential sex heterogeneity. Pearson correlation coefficients were also calculated to describe bivariate associations between Purpose in Life and each diabetes risk score.

All statistical analyses were conducted using IBM SPSS Statistics version 29, with statistical significance defined as *p* < 0.05.

## 3. Results

The analytical sample included 93,077 workers (55,900 men, 60.1%; 37,177 women, 39.9%), with a mean age of approximately 39.6 years. The majority of participants were classified in social class III (70.5%), and just over one third were current smokers (35.4%). Overall, 45.0% reported adherence to a Mediterranean dietary pattern, while 47.9% indicated regular engagement in physical activity. With respect to psychosocial status, 25.3% of individuals were categorized as having low Purpose in Life, 44.6% moderate, and 30.2% high.

Sex-stratified characteristics are presented in Table 1. Mean age was comparable between men and women; however, men exhibited higher values for anthropometric indices, blood pressure, triglycerides, fasting glucose, and most cardiometabolic risk markers, whereas women showed higher HDL cholesterol concentrations. Lifestyle patterns also differed by sex, with women more frequently reporting both Mediterranean diet adherence and regular physical activity. In addition, high Purpose in Life was substantially more prevalent among women, while low Purpose in Life was notably more common in men.

Table 2 summarizes the distribution of high-risk classifications derived from the three validated diabetes risk scores according to sociodemographic characteristics, lifestyle factors, and Purpose in Life categories, with results stratified by sex.

Variability in the proportion of participants classified as high risk across QDScore, FINDRISC, and CANRISK reflects differences in score design, target populations, prediction timeframes, and recommended cut-off values. Because each instrument was developed and calibrated within distinct healthcare contexts, differences in absolute prevalence are expected when applied to a Spanish working population. Notwithstanding these variations, a consistent gradient was observed across all three risk scores, with higher diabetes risk corresponding to lower levels of Purpose in Life.

Across all instruments, the prevalence of high estimated diabetes risk increased steadily with advancing age and was consistently greater among participants in lower occupational social classes, current smokers, individuals with poorer dietary adherence, and those reporting low levels of physical activity. In both men and women, reduced Purpose in Life was associated with a markedly higher prevalence of high-risk classification irrespective of the diabetes risk score used, indicating a clear and graded relationship at the descriptive level.

Bivariate analyses further supported these findings. Pearson correlation coefficients, presented in Supplementary Table S4, demonstrated inverse associations between Purpose in Life and each diabetes risk score (QDScore, FINDRISC, and CANRISK), consistent with the direction and magnitude of associations observed in the multivariable regression models.

Results from the multivariable logistic regression analyses (Table 3) indicate that lower levels of Purpose in Life were independently associated with an increased likelihood of being classified as high risk for type 2 diabetes across all three prediction tools. Relative to participants with high Purpose in Life, those categorized as moderate or low consistently exhibited elevated odds of high-risk status after adjustment for age, sex, occupational social class, smoking behavior, dietary adherence, physical activity, and body mass index.

Established sociodemographic and behavioral determinants—including advancing age, lower social class, current smoking, suboptimal diet, and physical inactivity—also showed significant associations with high diabetes risk, in line with existing epidemiological evidence and supporting the internal consistency of the regression models. Complete model specifications and covariate adjustments are provided in the footnote for Table 3.

Adjusted odds ratios (ORs) with corresponding 95% confidence intervals (CIs) were derived from multivariable logistic regression models contrasting moderate and low Purpose in Life with high Purpose in Life as the reference category. All models accounted for age, sex, occupational social class, body mass index, Mediterranean diet adherence, physical activity, and smoking status, and were estimated separately for QDScore, FINDRISC, and CANRISK high-risk classifications. The adjusted associations between Purpose in Life and elevated type 2 diabetes risk across the three instruments are summarized in Table 3.

**Figure 2:**
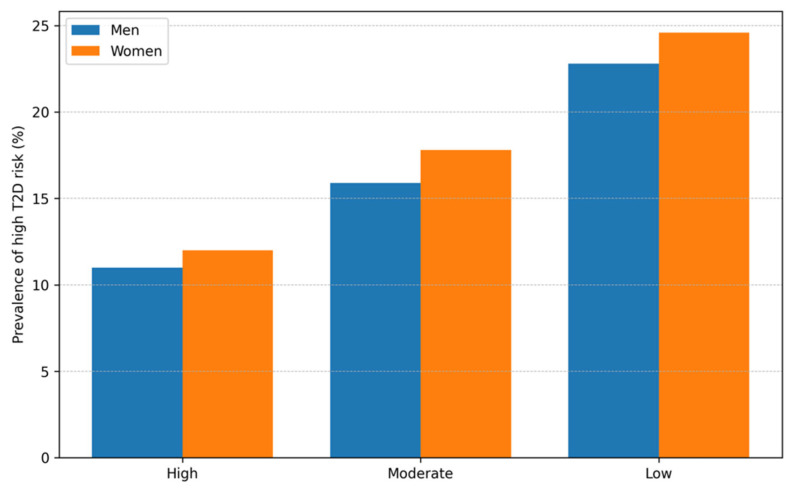
Prevalence of high type 2 diabetes risk (FINDRISC) by Purpose in Life category and sex.

Figure 2 illustrates the prevalence of high diabetes risk based on FINDRISC categories across Purpose in Life levels, stratified by sex. A pronounced gradient was evident in both men and women, with progressively greater prevalence of high-risk classification among individuals reporting lower Purpose in Life. Although absolute risk estimates differed between sexes, the overall pattern was consistent, indicating that reduced Purpose in Life is associated with a greater population-level burden of diabetes risk in both groups.

In Figure 2, bars indicate the proportion of participants classified as high risk according to FINDRISC across Purpose in Life categories (high, moderate, and low), presented separately for men and women.

Additional descriptive and crude analyses based on aggregated subgroup data are provided in the Supplementary Material (Tables S1–S3). Estimated counts of individuals classified as high diabetes risk by Purpose in Life category and sex are reported in Supplementary Table S1, derived from subgroup sizes and corresponding prevalence values.

Crude analyses using subgroup-level data demonstrated a clear graded association between decreasing Purpose in Life and increasing diabetes risk across all three prediction scores, with statistically significant linear trends observed in both sexes (Supplementary Table S2). No evidence of sex-related heterogeneity was detected in these crude associations for any of the risk instruments (Supplementary Table S3).

## 4. Discussion

In this cross-sectional analysis of over 93,000 employed adults, lower levels of Purpose in Life were consistently associated with greater estimated risk of type 2 diabetes as assessed by three validated and independent prediction tools. Given the observational and cross-sectional design, the results should be interpreted strictly as reflecting associations rather than causal effects. Participants categorized as having moderate or low Purpose in Life exhibited significantly higher odds of high-risk classification compared with those reporting high levels, even after accounting for sociodemographic factors and key lifestyle determinants. Importantly, the direction and strength of these associations were similar across QDScore, FINDRISC, and CANRISK, reinforcing the stability of the findings across distinct risk prediction frameworks.

**Figure 3:**
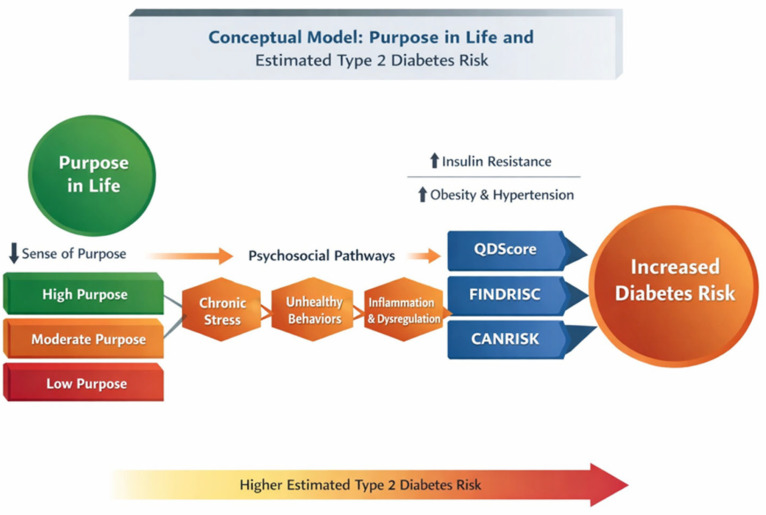
Conceptual model.

Figure 3 presents a conceptual model outlining potential behavioral and biological pathways linking Purpose in Life with estimated diabetes risk. Lower perceived purpose may contribute to impaired motivational regulation and increased stress burden, thereby promoting adverse health behaviors and psychophysiological dysregulation. These mechanisms could, in turn, influence cardiometabolic risk factors incorporated within established diabetes risk scores.

A conceptual framework summarizing the potential behavioral and biological pathways linking Purpose in Life with diabetes risk is presented in Figure 3.

An important feature of the present findings is the presence of a clear dose–response relationship. Across all three risk instruments and in both men and women, estimated diabetes risk rose progressively as levels of Purpose in Life declined. This graded pattern reduces the likelihood of a chance association and is consistent with longitudinal evidence linking stronger life purpose to lower rates of chronic disease and mortality [27,28,29,30]. Previous cohort studies have similarly reported that individuals with higher Purpose in Life experience fewer cardiovascular events, reduced stroke incidence, and lower all-cause mortality, supporting the notion that purpose functions as a broadly protective psychosocial resource [31].

The current results also align with an expanding literature demonstrating that positive psychosocial attributes are associated with more favorable cardiometabolic profiles and healthier aging trajectories [32,33,34]. Higher Purpose in Life has been related to beneficial health behaviors, including greater physical activity, improved sleep, healthier dietary choices, and lower smoking prevalence [33]. Notably, in the present analyses, the association between Purpose in Life and diabetes risk persisted after adjustment for major lifestyle factors, indicating that behavioral pathways account for part—but not the entirety—of the observed relationship, in line with findings from prior mediation-focused cohort studies [35].

Several plausible mechanisms may explain these findings. From a behavioral perspective, higher Purpose in Life may enhance motivational regulation, future-oriented decision-making, and sustained adherence to health-protective behaviors [36]. From a psychobiological standpoint, greater purpose and psychological well-being have been associated with lower chronic stress burden, healthier diurnal cortisol profiles, reduced inflammatory activity, and more favorable cardiometabolic biomarker patterns. Chronic stress and dysregulated hypothalamic–pituitary–adrenal axis activity are well-established contributors to insulin resistance and metabolic dysfunction [37,38,39]. These converging behavioral and biological pathways provide a coherent explanatory framework for the observed associations.

From a clinical and public health perspective, the findings suggest that psychosocial well-being—and specifically Purpose in Life—may represent a relevant dimension in cardiometabolic risk profiling. There is increasing recognition that psychosocial factors contribute meaningfully to chronic disease risk and prevention strategies [40,41]. While current diabetes risk scores focus primarily on demographic, anthropometric, and behavioral factors, psychosocial constructs could contribute additional stratification value, particularly in preventive and occupational health contexts. Brief validated measures of purpose and well-being are feasible to administer and have shown predictive value for long-term health outcomes in population studies [42,43,44,45,46].

Although prior prospective studies have reported associations between Purpose in Life and subsequent health outcomes, the present analyses are cross-sectional and all variables were measured concurrently. Accordingly, temporal ordering between exposure and outcome cannot be established and reverse causation cannot be excluded. The findings should therefore be interpreted strictly as associative. Longitudinal studies will be required to determine whether Purpose in Life independently predicts future diabetes incidence beyond established risk factors.

## 5. Strengths and Limitations

This study has several important strengths. First, the very large sample size provided high statistical precision, narrow confidence intervals, and stable effect estimates across multiple subgroup analyses, which increases the reliability of observed associations in population-based research [47]. Second, diabetes risk was assessed using three independent and widely validated risk scores—QDScore, FINDRISC, and CANRISK—allowing cross-instrument verification of the associations and reducing the likelihood that findings are driven by score-specific measurement properties [48,49,50]. The use of multiple validated instruments is considered a robust methodological strategy to enhance construct validity and reproducibility. Third, multivariable models included adjustment for a broad set of sociodemographic and lifestyle factors known to influence diabetes risk, consistent with current epidemiologic modeling recommendations [51]. Fourth, the presence of consistent dose–response gradients across Purpose in Life categories and across sexes strengthens internal validity and supports biological and behavioral plausibility [52].

Several limitations warrant consideration. First, the cross-sectional nature of the study prevents causal interpretation and does not permit establishment of temporal ordering between exposure and outcome, a constraint inherent to observational designs of this type. Accordingly, the title and interpretations have been framed to reflect associations only. In addition, although participants underwent routine clinical and laboratory evaluation including fasting glucose measurement, the presence of undiagnosed type 2 diabetes or intermediate hyperglycemia cannot be completely excluded. This limitation is inherent to large population-based cross-sectional studies relying on risk prediction tools rather than diagnostic criteria. Such potential misclassification would likely bias associations toward the null rather than inflate observed effects.

Alcohol consumption was not available in this dataset, which may have resulted in residual confounding by unmeasured lifestyle factors. This limitation is inherent to the standardized occupational health surveillance protocol used and should be considered when interpreting the results.

Given the cross-sectional design, temporal ordering between exposure and outcome cannot be established, and reverse causation cannot be excluded. Bidirectional relationships between psychosocial factors and health status therefore cannot be fully ruled out [53]. Purpose in Life and lifestyle variables were obtained through self-report, which introduces the possibility of reporting inaccuracies and social desirability effects, a well-recognized source of bias in behavioral epidemiology [54]. In addition, diabetes risk scores are predictive tools rather than clinical diagnoses and therefore involve some degree of misclassification error, although all three instruments used have demonstrated acceptable calibration and discrimination in prior validation studies [55]. Supplementary crude analyses based on aggregated subgroup data should be interpreted as supportive rather than confirmatory, since aggregated analyses may introduce ecological approximation bias [56]. Finally, as in all observational research, residual confounding due to unmeasured or imperfectly measured variables cannot be completely ruled out [57].

## 6. Final Interpretive Closing Paragraph

Taken together, these findings support the hypothesis that Purpose in Life is not only a psychological construct but also a meaningful correlate of cardiometabolic risk profiles at the population level. The consistency of the associations across three validated diabetes risk scores, the presence of dose–response gradients, and the robustness across sociodemographic and behavioral strata strengthen the credibility of the observed relationship. While causality cannot be inferred from cross-sectional data, the results provide a strong rationale for prospective and interventional studies to examine whether enhancing purpose and related psychosocial resources may contribute to more effective diabetes risk prevention strategies. Integrating psychosocial dimensions into cardiometabolic risk frameworks may represent a valuable next step in precision prevention and workplace health promotion.

## 7. Conclusions

Lower Purpose in Life is consistently associated with higher estimated type 2 diabetes risk across three validated risk scores in a large working population. The presence of dose–response gradients and cross-instrument consistency supports the robustness of the association. Incorporating psychosocial dimensions such as Purpose in Life into preventive and occupational health frameworks may enhance cardiometabolic risk identification and intervention strategies. Prospective studies are needed to evaluate causal pathways and intervention potential.

## Figures and Tables

**Table 1:** Baseline characteristics of the study population by sex.

	Men *n* = 55,900	Women *n* = 37,177	Total (*n* = 93,077)		
Variables	Mean (SD)	Mean (SD)	Mean (SD)	*p*-Value	Cohen’s d
Age (years)	39.8 (10.3)	39.3 (10.2)	39.6 (10.3)	<0.001	0.05
Height (cm)	174.0 (7.0)	161.2 (6.6)	169.0 (6.8)	<0.001	1.87
Weight (kg)	81.2 (13.9)	65.4 (13.2)	74.9 (13.4)	<0.001	1.16
Waist (cm)	87.7 (9.1)	73.9 (7.9)	82.1 (8.4)	<0.001	1.60
Hip (cm)	100.1 (8.4)	97.3 (8.9)	99.0 (8.8)	<0.001	0.33
Systolic BP (mm Hg)	124.3 (14.9)	114.5 (15.0)	120.3 (14.6)	<0.001	0.66
Diastolic BP (mm Hg)	75.4 (10.6)	69.7 (10.4)	73.1 (10.5)	<0.001	0.54
Cholesterol (mg/dL)	195.9 (38.8)	193.5 (36.4)	194.9 (37.8)	<0.001	0.06
HDL-c (mg/dL)	51.0 (7.1)	53.8 (7.7)	52.2 (7.5)	<0.001	−0.38
LDL-c (mg/dL)	120.5 (37.7)	122.1 (37.0)	121.2 (37.4)	<0.001	−0.04
Triglycerides (mg/dL)	123.7 (87.7)	88.5 (47.2)	109.6 (61.2)	<0.001	0.47
Glucose (mg/dL)	88.1 (13.0)	84.1 (11.5)	86.5 (12.4)	<0.001	0.32
Variables	***n*** **(%)**	***n*** **(%)**		***p*** **-value**	
18–29 years	9956 (17.8)	7193 (19.3)	17,149 (18.4%)	<0.001	
30–39 years	18,525 (33.1)	12,319 (33.1)	30,844 (33.1%)		
40–49 years	16,632 (29.8)	11,035 (29.7)	27,667 (29.7%)		
50–59 years	9062 (16.2)	5669 (15.2)	14,731 (15.8%)		
60–69 years	1725 (3.1)	961 (2.6)	2686 (2.9%)		
Social class I	2964 (5.3)	2587 (7.0)	5551 (6.0%)	<0.001	
Social class II	9702 (17.4)	12,197 (32.8)	21,899 (23.5%)		
Social class III	43,234 (77.3)	22,393 (60.2)	65,627 (70.5%)		
Smokers	20,659 (37.0)	12,262 (33.0)	32,921 (35.4%)	<0.001	
Yes Mediterranean diet	22,838 (40.9)	19,096 (51.4)	41,934 (45.0%)	<0.001	
Yes physical activity	25,285 (45.2)	19,337 (52.0)	44,622 (47.9%)	<0.001	
Purpose in Life low	19,071 (34.1)	4432 (11.9)	23,503 (25.3%)	<0.001	
Purpose in Life moderate	27,707 (49.6)	13,774 (37.0)	41,481 (44.6%)		
Purpose in Life high	9122 (16.3)	18,971 (51.0)	28,093 (30.2%)		

BP, Blood pressure. HDL, High density lipoprotein. LDL, Low density lipoprotein. SD, Standard deviation. Cohen’s d is reported for continuous variables as a measure of effect size (small = 0.2, medium = 0.5, large = 0.8).

**Table 2:** Distribution of diabetes risk indices according to sociodemographic, behavioral factors, and Purpose in Life.

		QDScore > 3		FINDRISC High		CANRISK High
Men	*n*	%	*n*	%	*n*	%
18–29 years	6095	6.7	9956	0.4	9956	1.4
30–39 years	18,525	7.5	18,525	1.1	18,525	2.8
40–49 years	16,632	9.9	16,632	3.8	16,632	11.4
50–59 years	9062	12.5	9062	8.2	9062	33.4
60–69 years	1725	13.9	1725	15.3	1725	51.7
Social class I	2886	7.0	2964	2.9	2964	5.6
Social class II	9256	7.5	9702	3.3	9702	7.4
Social class III	39,897	9.7	43,234	3.4	43,234	12.9
Smokers	18,959	9.8	20,659	3.6	20,659	12.6
Non-smokers	33,080	8.8	35,241	3.0	35,241	9.8
Yes Mediterranean diet	20,125	4.9	22,838	1.6	22,838	7.1
Non-Mediterranean diet	31,914	10.5	33,062	4.2	33,062	15.8
Yes physical activity	22,358	3.2	25,285	0.9	25,285	4.0
Non-physical activity	29,681	14.5	30,615	5.3	30,615	18.9
Purpose in Life low	18,984	13.9	19,071	4.8	19,071	15.8
Purpose in Life moderate	26,929	9.2	27,707	3.5	27,707	10.9
Purpose in Life high	6126	5.6	9122	2.2	9122	7.7
Women	***n***	**%**	***n***	**%**	***n***	**%**
18–29 years	4612	8.4	7193	0.2	7193	0.2
30–39 years	12,319	10.1	12,319	0.8	12,319	0.3
40–49 years	11,035	11.6	11,035	2.5	11,035	3.0
50–59 years	5669	12.3	5669	7.3	5669	11.4
60–69 years	961	14.4	961	12.8	961	22.5
Social class I	2488	6.6	2587	1.5	2587	0.9
Social class II	11,494	7.4	12,197	1.9	12,197	1.2
Social class III	20,610	13.8	22,393	3.1	22,393	4.9
Smokers	11,269	11.4	12,262	2.8	12,262	4.0
Non-smokers	23,323	10.6	24,915	1.8	24,915	1.8
Yes Mediterranean diet	17,331	7.2	19,096	1.6	19,096	2.1
Non-Mediterranean diet	17,261	15.1	18,081	3.5	18,081	4.8
Yes physical activity	17,547	4.5	19,337	0.8	19,337	1.0
Non-physical activity	17,045	18.0	17,840	4.3	17,840	5.9
Purpose in Life low	4322	16.5	4432	6.6	4432	4.9
Purpose in Life moderate	13,469	10.3	13,774	4.0	13,774	3.3
Purpose in Life high	16,801	7.1	18,971	2.9	18,971	2.0

FINDRISC, Finnish Diabetes Risk Score. CANRISK, Canadian Diabetes Risk Questionnaire. QDScore, Q Diabetes Score. *p* < 0.001 in all cases. High-risk categories were defined according to standard recommended cut-off values for each instrument (QDScore, FINDRISC, and CANRISK). Reported values represent the number and percentage of participants exceeding these thresholds.

**Table 3:** Multivariable associations between sociodemographic, lifestyle, psychosocial factors, and type 2 diabetes risk.

	QDScore > 3	FINDRISC High	CANRISK High
	OR (95% CI)	OR (95% CI)	OR (95% CI)
Women	1	1	1
Men	0.80 (0.77–0.84)	1.95 (1.80–2.11)	1.88 (1.74–2.03)
18–29 years	1	1	1
30–39 years	1.19 (1.15–1.23)	1.36 (1.19–1.54)	1.60 (1.44–1.77)
40–49 years	1.49 (1.41–1.57)	1.64 (1.43–1.86)	3.40 (3.04–3.77)
50–59 years	1.86 (1.73–1.99)	3.01 (2.54–3.49)	8.72 (7.62–9.83)
60–69 years	2.40 (2.20–2.61)	5.61 (4.15–7.08)	10.09 (8.29–11.90)
Social class I	1	1	1
Social class II	1.23 (1.18–1.29)	1.19 (1.14–1.25)	2.79 (2.55–3.03)
Social class III	1.88 (1.70–2.07)	1.49 (1.36–1.63)	3.73 (3.14–4.33)
Non-smokers	1	1	1
Smokers	1.22 (1.18–1.27)	1.29 (1.20–1.39)	1.20 (1.15–1.26)
Yes Mediterranean diet	1	1	1
Non-Mediterranean diet	2.69 (2.48–2.91)	2.50 (2.08–2.92)	3.55 (2.79–4.32)
Yes physical activity	1	1	1
Non-physical activity	4.14 (3.65–4.64)	4.43 (3.64–5.23)	6.32 (5.07–7.58)
Purpose in Life high	1	1	1
Purpose in Life moderate	1.60 (1.49–1.71)	1.78 (1.59–1.98)	1.85 (1.62–2.09)
Purpose in Life low	2.38 (2.19–2.57)	2.49 (2.08–2.89)	2.79 (2.50–3.09)

FINDRISC, Finnish Diabetes Risk Score. CANRISK, Canadian Diabetes Risk Questionnaire. QDScore, Q Diabetes Score. OR, Odds ratio. CI, Confidence Interval. *p* < 0.001 in all cases. Models adjusted for age, sex, occupational social class, smoking status, Mediterranean diet adherence, physical activity, and body mass index.

## Data Availability

The datasets generated and analyzed during the current study are available from the corresponding author upon reasonable request and are stored under the supervision of the institutional Data Protection Officer at ADEMA University School.

## Supplementary Materials

The following supporting information can be downloaded at: https://www.mdpi.com/article/10.3390/medsci14010113/s1, Table S1: Estimated cases of high type 2 diabetes risk according to Purpose in Life category and sex; Table S2: Crude odds ratios for high diabetes risk according to Purpose in Life category; Table S3: Sex heterogeneity tests for crude associations between Purpose in Life and diabetes risk. Table S4: Pearson correlation coefficients.
