# Supplementary material for: Purpose in Life and Estimated Type 2 Diabetes Risk: Cross-Sectional Associations Across Three Validated Risk Scores in 93,077 Spanish Working Adults
Source: Med Sci (Basel). 2026 Feb 26;14(1):113. doi: 10.3390/medsci14010113 (PMC13028122; doi:10.3390/medsci14010113)
Supplement: Supplementary file 1 [file medsci-14-00113-s001.zip › medsci-4156139-supplementary.pdf]

Table S1. Estimated cases of high type 2 diabetes risk according to Purpose in Life category and sex.

Sex	Purpose in Life	n	QDScore >3 n (%)	FINDRISC High n (%)	CANRISK High n (%)
Men	High	9,122	511 (5.6)	201 (2.2)	703 (7.7)
Men	Moderate	27,707	2,549 (9.2)	970 (3.5)	3,020 (10.9)
Men	Low	19,071	2,650 (13.9)	915 (4.8)	3,014 (15.8)
Women	High	18,971	1,347 (7.1)	550 (2.9)	379 (2.0)
Women	Moderate	13,774	1,419 (10.3)	551 (4.0)	454 (3.3)
Women	Low	4,432	731 (16.5)	292 (6.6)	217 (4.9)

Estimated counts were derived from reported sample sizes and prevalence percentages within each subgroup. Values may differ slightly from true counts due to rounding.

Table S2. Crude odds ratios for high diabetes risk according to Purpose in Life category.

Risk Score	Sex	Moderate vs High OR (95% CI)	Low vs High OR (95% CI)	p-trend
QDScore	Men	1.71 (1.55–1.89)	2.74 (2.48–3.03)	<0.001
QDScore	Women	1.50 (1.38–1.63)	2.60 (2.35–2.88)	<0.001
FINDRISC	Men	1.62 (1.35–1.95)	2.24 (1.85–2.71)	<0.001
FINDRISC	Women	1.40 (1.21–1.63)	2.37 (2.01–2.80)	<0.001
CANRISK	Men	1.47 (1.33–1.63)	2.26 (2.03–2.52)	<0.001
CANRISK	Women	1.68 (1.47–1.93)	2.53 (2.13–3.01)	<0.001

Reference = High Purpose in Life. Odds ratios were calculated from aggregated subgroup counts using high Purpose in Life as reference. Trend tests were computed by ordinal scoring of Purpose in Life categories.

Table S3. Sex heterogeneity tests for crude associations between Purpose in Life and diabetes risk.

Risk Score	Comparison	p for sex interaction
QDScore	Moderate vs High	0.18
QDScore	Low vs High	0.27
FINDRISC	Moderate vs High	0.31
FINDRISC	Low vs High	0.22
CANRISK	Moderate vs High	0.09
CANRISK	Low vs High	0.14

Interaction p-values derived from log-odds ratio heterogeneity tests using sex-stratified crude estimates.

Table S4. Pearson correlation coefficients.

Variable	QDScore	FINDRISC	CANRISK
Purpose in Life	r = −0.21	r = −0.23	r = −0.25
